# Is integration of healthy lifestyle promotion into primary care feasible? Discussion and consensus sessions between clinicians and researchers

**DOI:** 10.1186/1472-6963-8-213

**Published:** 2008-10-14

**Authors:** Gonzalo Grandes, Alvaro Sanchez, Josep M Cortada, Laura Balague, Carlos Calderon, Arantza Arrazola, Itziar Vergara, Eduardo Millan, the "Prescribe Vida Saludable" group

**Affiliations:** 1 Primary Care Research Unit of Bizkaia, Basque Health Service (Osakidetza), Bilbao, Spain; 2 Deusto Health Centre, Basque Health Service (Osakidetza), Bilbao, Spain; 3 Renteria Health Centre, Basque Health Service (Osakidetza), Renteria, Spain; 4 Alza Health Centre, Basque Health Service (Osakidetza), Donostia-San Sebastian, Spain; 5 Health Plan Service in Gipuzkoa. Department of Health of the Basque Government; 6 O+berri Institute, Basque Foundation for Health Innovation and Research, Sondika, Spain; 7 Cruces Hospital, Basque Health Service (Osakidetza), Barakaldo, Spain; 8 "Prescribe Vida Saludable" group, Bilbao, Spain

## Abstract

**Background:**

The adoption of a healthy lifestyle, including physical activity, a healthy diet, moderate alcohol consumption and abstinence from smoking, is associated with a major decrease in the incidence of chronic diseases and mortality. Primary health-care (PHC) services therefore attempt, with rather limited success, to promote such lifestyles in their patients. The objective of the present study is to ascertain the perceptions of clinicians and researchers within the Basque Health System of the factors that hinder or facilitate the integration of healthy lifestyle promotion in routine PHC setting.

**Methods:**

Formative research based on five consensus meetings held by an expert panel of 12 PHC professionals with clinical and research experience in health promotion, supplied with selected bibliographic material. These meetings were recorded, summarized and the provisional findings were returned to participants in order to improve their validity.

**Results:**

The Health Belief Model, the Theory of Planned Action, the Social Learning Theory, "stages of change" models and integrative models were considered the most useful by the expert panel. Effective intervention strategies, such as the "5 A's" strategy (assess, advise, agree, assist and arrange) are also available. However, none of these can be directly implemented or continuously maintained under current PHC conditions. These strategies should therefore be redesigned by adjusting the intervention objectives and contents to the operation of primary care centres and, in turn, altering the organisation of the centres where they are to be implemented.

**Conclusion:**

It is recommended to address optimisation of health promotion in PHC from a research perspective in which PHC professionals, researchers and managers of these services cooperate in designing and evaluating innovative programs. Future strategies should adopt a socio-ecological approach in which the health system plays an essential role but which nevertheless complements other individual, cultural and social factors that condition health. These initiatives require an adequate theoretical and methodological framework for designing and evaluating complex interventions.

## Background

Lifestyle changes can result in substantial health benefits given that a sedentary lifestyle, an unhealthy diet, smoking and alcohol abuse are the main causes of morbidity and mortality in industrialised countries [1]. Such unhealthy behaviours do not occur in isolation, but tend to be clustered in the same individuals [2,3]. It has been estimated that only 9% of the Spanish population aged 18–64 years routinely performs physical activity, follows a healthy diet, does not smoke and does not drink in excess, with 50% of the population combining at least two of these risk behaviours and 18% at least three. All four risk behaviours are found in around 3% of the population [3]. Such lifestyles result in unnecessary suffering and a disproportionate and avoidable burden on health systems. The WHO estimates, for instance, that approximately 80% of cardiovascular diseases, 90% of type 2 diabetes and 30% of all cancers could be prevented if the population followed a healthy diet, engaged in an adequate level of physical activity and ceased smoking [4]. Recent studies attribute a potential reduction of overall mortality by approximately 60% and a 14-year increase in life expectancy to adoption of a healthy lifestyle [5,6].

These figures represent a call to action, and primary health-care (PHC) professionals are therefore being recommended to include behaviour-changing advice as part of their standard clinical practice [7-9]. The long-term nature of PHC provides family physicians and nursing staff with multiple opportunities over time for counselling and influencing the risk factors and healthy behaviour of the general population. PHC is the most accessible level of care, attended by the majority of the population: almost 95% of people attend their health centre at least once in a five-year period. A unique situation prevails in PHC services, namely that they reach practically the whole community on a one-to-one basis.

The problem lies in that helping people change their lifestyle behaviours is not an easy task. Individual behaviour is dictated by multiple personal, institutional and environmental factors that operate and interact at individual, interpersonal and community levels [10]. The complexity involved in approaching this difficult task within a context of work overload and shortage of time and training, combined with the lack of knowledge about how individuals may be influenced from this socio-ecological perspective, has meant that the results of health promotion programs implemented in PHC remain relatively modest [2,11-13]. As a result, health promotion is far from being an integral part of routine clinical practice in PHC [13], and evidence concerning the effectiveness of interventions, strategies, or programs intended to optimise preventative and health-promotion services is still limited and inconclusive [14,15].

The objective of this article is to summarise the perceptions of an expert panel in clinical practice and research in the field of health promotion in PHC, regarding the factors hindering or favouring the integration and outcomes of healthy lifestyle promotion in routine PHC within the Basque Health System in Spain. The definition of PHC includes at the very least health education for individuals and the whole community [16]. The terms of health education and health promotion are closely related and overlapped. Health education focuses on strategies aimed at increasing knowledge, motivation and skills and at changing behaviours in order to improve health, taking into account the underlying social, economic and environmental conditions impacting on health, including the health-care system. In this project we link health education to a wide range of actions similar to those outlined in the Ottawa Charter for health promotion: build healthy public policy, create supportive environments for health, strengthen community action for health, develop personal skills and re-orient health services [17]. We therefore use the term "health promotion" in a broad sense that covers health education and other related organizational, economic and environmental tools for promoting the behaviour of individual groups or communities conducive to health.

## Methods

A formative research project was designed based on five structured discussion and consensus meetings held by an expert panel. We adopted a consensus methodology because scientific evidence regarding the most applicable theoretical models of health promotion, the effectiveness of multiple risk factors interventions and the factors associated with the integration of health promotion in primary care is limited, inconclusive or non-existent [2,7,10,12,14,15]. Among other consensus methods available we discarded the Delphi technique because it diminishes the positive aspects of interaction at face-to-face meetings. An adapted nominal-group technique was used to promote debate, interaction and to generate a wide range of ideas. The expected outcome was an appropriate definition of the problem of health promotion integration in PHC, in order to identify opportunities for innovative interventions that could result in meaningful improvements in preventive care delivery.

In January 2006, the Primary Care Research Unit of Bizkaia convened a group of 12 health-care professionals with a special interest and clinical and research experience in integrating health promotion into primary health-care. This multidisciplinary group consisted of family physicians and nurses, epidemiologists, specialists in preventive medicine and public health, specialists in health education, psychologists and sociologists [18]. PHC in Spain is mostly publicly funded and run by the Governments of the Autonomous Communities. Health professionals have a status similar to that of civil servants and work in team practices called primary care centres, which attend to the population living in a defined geographical area. The Spanish PHC system provides universal free health-care with an extremely high level of accessibility. Every Spanish citizen is assigned to a unique general practitioner who acts as a gatekeeper to the more specialized levels of the system. In the Basque Country, on average, approximately 2,000 inhabitants are assigned to every general practitioner, who attend 30 patients per day during seven hours of scheduled practice, including home visits. The functions formally assigned to primary-care teams include health promotion, health education and preventative services; diagnostic and therapeutic procedures; medical, nursing and community care; and rehabilitation and palliative care [19].

No ethics committee approval was required in this study as it was commissioned to the Primary Care Research Unit of Bizkaia by the Health Department of the Basque Government. In this kind of commissioned project the Health Department itself determines the objectives, methods, research team composition and expected outcomes of the study. Informed consent was also unnecessary as participants were 12 experts who were directly informed about the study objectives by the Head of the Research Unit and who agreed to take part in the five discussion and consensus meetings.

### Structured discussion and consensus meetings

The expert panel held five sessions between January and May 2006, each of which lasted approximately two hours, aimed at answering the following questions:

1st session.- *What theories or theoretical models are most commonly used in the health education and promotion area to understand people's behaviour and encourage changes to risky lifestyle behaviours? Which of these theories or theoretical models are most applicable to designing and evaluating programs aimed at provoking changes in lifestyle behaviours in PHC?*

2nd session.- *What is the scientific evidence regarding the value of constructs and variables of theoretical models for changing the following risky lifestyle behaviours: smoking, alcohol, diet and a sedentary lifestyle? Which variables are most useful for designing healthy lifestyle promotion programs in PHC?*

3rd session.- *How effective are intervention strategies targeting smoking, alcohol, diet and sedentary behaviour in a PHC setting? Which key components of interventions would be useful and effective for integration into programs to promote healthy lifestyle behaviours?*

4th session.- *To what extent is it important to address such lifestyle behaviours in PHC? Which factors in our health system facilitate or hinder activities for promoting healthy lifestyle behaviours and implementation of strategies to address and manage such activities?*

5th session.- *How can intervention strategies that have been shown to be effective be implemented in PHC in our national health system? What changes would be required in PHC offices and centres, as well as in the organisation of health services, to facilitate implementation of such strategies?*

The specific topics were given to the group members before each session, and selected support materials in line with the session objectives were provided along with a summary of the previous session and the objectives for the current session. At the meeting, one of the researchers acted as a facilitator and, after requesting authorization to record the meetings whilst guaranteeing that the information would be treated confidentially, introduced the questions to be addressed. Each of the participants briefly presented each topic while all other members prepared relevant issues to be raised after all topic presentations. A second researcher acted as an observer, noting down the most relevant ideas. Once issues had been raised, ideas on which there was agreement and disagreement were identified until all the contributions from within each meeting had been exhausted.

The complete sessions were recorded using two digital recorders to facilitate the process of summarising their content and provisional results, which were subsequently sent to each of the group members for approval, together with the notification of the date of the next session and the session objective and agenda. In order to ensure the accuracy of the results and consensus in the resulting final document, a draft circulation system was organised to allow collaborating professionals to verify the validity of the document or make any clarifications or changes.

### Literature review

Bibliographic materials were used to support these meetings. In order to select concise scientific documentation to facilitate and enrich the discussion, two rapid, non-exhaustive reviews were made on theoretical models and the effectiveness of intervention strategies for modifying lifestyles in PHC setting. The first search targeted original studies indexed in MEDLINE between 1996 and 2006 which assessed interventions to modify a sedentary lifestyle, inadequate diet, alcohol abuse and smoking, conducted on healthy adult patients in PHC. We focused our attention on seminal theories that can help primary-care practitioners conceptualize the complex nature of healthy behaviours and identified studies in which the mediating or modulating effect of variables derived from theoretical health-behaviour models had been analyzed. Among the numerous existing theories, the search was restricted to those used most frequently, and supported empirically, in the field of primary care (see Table 1) [10,20]. Cross-sectional studies, studies where part of the intervention had been performed by non-PHC professionals and studies with poor quality methodology in terms of the description of the measurement processes and presentation of the results, or those with a follow-up period shorter than three months, were excluded.

**Table 1:** Summary of the main theoretical models of behavioural change in primary care

**Theory/Model**	**Description**	**Key variables and constructs**
*Individual level: knowledge, beliefs, attitudes, personality traits, past experiences and change processes*		

		

Health Belief Model [17,18]	Healthy behaviour is the result of perception of disease susceptibility and severity, perception of the benefits of the behaviour required for disease avoidance or management, exposure to stimuli promoting the action, and personal confidence in the capacity to successfully implement the behaviour.	Perceived susceptibility Perceived severity Perceived benefits and barriers Cues to action Self-efficacy
Theory of Reasoned/Planned Action [19-21]	Behavioural intention determines the performance of a given behaviour through the influence exerted by beliefs, attitudes, subjective norms and perceived control on intention and behaviour itself.	Behavioural intention Subjective norms Attitude toward behaviour Perceived behavioural control
Information Processing Model [22]	The capacity of the person to understand and react to information and communication sources influences his/her behaviour.	Who provides the information How information is created, transmitted, received and assimilated
Transtheoretical Model of Stages of Change [23]	Willingness or intention to change behaviour varies among individuals and within an individual over time. Relapse is a common event and part of the change process.	Stages of change: (1) Precontemplation, (2) Contemplation, (3) Preparation, (4) Action, (5) Maintenance. Change processes: Cognitive and behavioural; Self-efficacy
Precaution adoption process [24,25]	Adoption of a new behaviour requires a process, consisting of 7 stages or steps, from ignorance of the problem, through the decision to perform the action, to the final change in behaviour.	Stages: (1) No risk awareness; (2) Aware of risk, but considers oneself not susceptible to it; (3) Decision-making process, which may be: (4) No action; (5) Ready for action; (6) Action; (7) Maintenance

*Interpersonal level: role of environment and social support network*		

		

Operating Learning Model [26]	The probability of performing a behaviour is dictated by the history of consequences (environmental changes, stimuli) contingent to its performance. Behaviours should be defined based on the variables that control them: antecedents (stimulus situation prior to behaviour performance) and consequences (change in environment or stimulus situation immediate to behaviour performance).	Antecedent stimuli; Consequences; Reinforcement principle (positive or negative reinforcement); Principle of punishment (positive or negative punishment); Stimulus control; Reinforcing cultural contingencies
Social Learning or Social-Cognitive Model [27,28]	Behaviour is dictated by dynamic interaction of personal factors, environmental influences and behaviour: reciprocal determinism.	Observational learning Outcome and self-efficacy expectations Behavioural capacity; Reinforcement
Self-regulation models [29]	Effectiveness in long-term behavioural change depends on the degree of control the individual has on his/her process of change.	Self-management skills; Self-monitoring; Self-evaluation; Self-reinforcement
Interpersonal and social support theories [30]	Effective interpersonal communication between the provider and patient, taking into account the significance of the environment surrounding the individual, is essential for the change to occur.	Informative support Emotional support Environment collaboration

*Community level*		

		

Community-based intervention approach [31]	Community well-being may be promoted by identification of common problems and objectives, resource mobilisation and development and implementation of strategies to reach such collective objectives, including the creation of structures and policies supporting healthy practices and lifestyles.	

The second search focused on systematic reviews and meta-analyses indexed in MEDLINE and the Cochrane Library between 1996 and 2006 which assessed the effectiveness of medical advice in PHC about modification of the above lifestyle behaviours in healthy adult patients. Papers lacking systematic search processes, study selection based on the quality of the methodology, or a summary of results, were excluded. A selection was made of the most relevant studies that provide evidence about effective intervention strategies or include components with encouraging results, and whose findings and conclusions were relevant for PHC services, with the purpose of promoting the discussion sessions. Detailed information about both the methodological characteristics and the contents and results of interventions was taken from each of the selected papers and displayed in summary tables of evidence.

## Results

### Theoretical models and constructs useful for promoting healthy lifestyle behaviours

The theoretical models summarised in Table 1 were identified and discussed [21-37]. These models attempt to explain healthy behaviours and define operational variables whose manipulation using intervention strategies could lead to a change in behaviour [38,39]. The expert panel considered the Health Belief Model, which is most widely supported by use in multiple programs over time, the Theory of Planned Behaviour and the Social Learning Theory, which introduce the dimension of social and environmental influence, and models describing the staged change process in a schematic and operational way, to be of particular value for the design and evaluation of interventions in the PHC setting. While scientific evidence is poor, it was noted that some variables in these models play a mediating role in behavioural change. These variables include the social support network, perceived benefits and barriers and change processes (for physical activity) [40-45]; perceived benefits, perceived efficacy, anticipated regret, social support and understanding of nutritional recommendations (for diet) [46-51]; and dependence level and prior partially successful attempts, related with intention to change and self-efficacy (for smoking) [52-54]. The group greatly appreciated integrative initiatives such as that proposed by Fishbein [37], which attempts to combine many of the components of prior models into a single model.

### Effective intervention strategies for addressing risky lifestyle behaviours in PHC

All expert group members considered integration of health promotion within PHC activities to be necessary and potentially feasible. There is strong evidence supporting the effectiveness of brief counselling for achieving smoking cessation and reducing alcohol intake [55-62]. Counselling about physical activity achieves little result while prescription of a physical activity plan achieves more relevant results, though such results wane over time [12,63-69]. Intensive medical counselling interventions induce small to moderate changes in dietary components [70-74]. These effective interventions are perfectly adapted to the 5-A's intervention strategy: assess, advise, agree, assist and arrange follow-up [75]. This was considered to be the most useful strategy among those used in PHC because it is simpler, may be applied on an individual basis, requires less time and training, and scientific evidence is available about its effectiveness in the general population.

In order to achieve greater effectiveness, it was considered desirable to investigate new ways of integrating all three intervention strategies considered – the 5-A's, motivational interviewing and a community-based approach – even though the reviewed meta-analyses and studies report equivocal results about the effectiveness of the latter two strategies [76-80]. A simultaneous approach to multiple risk factors has only been evaluated and is relatively effective for secondary prevention interventions in patients with cardiovascular disease, diabetes, or a high risk of disease [2,81]. Interventions with multiple components, which combine medical counselling with behavioural interventions and resources outside of the health system, appear to be the most promising (Table 2).

**Table 2:** Intervention components associated wit modification of lifestyle behaviours in primary care setting

Physical activity	Combining advice from the family physician with behavioural interventions such as: goal-setting for the patient, written prescriptions and physical activity regimens adapted to the individual, multiple follow-up contacts by telephone or mail, performed by trained staff, linking or referring to physical activity resources in the community or to exercise programs.
Diet	Combining advice from the family physician with assistance systems at the primary care centre such as advice algorithms, warning or reminding mechanisms, interactive communication media (tailored emails, telephone advice) and group interventions. At the patient level, goal-setting, provision of feedback and behavioural reinforcement, education on nutrition and diet, support materials such as food acquisition and preparation guides, self-monitoring techniques, training to overcome barriers in healthy food selection, social support networks or resources.
Smoking	Counselling or therapeutic interventions for motivated patients related to problem solving, skills training, relapse prevention, stress management, multiple follow-up contacts and intervention in the smoker's environment to increase social support and enhance the effect of brief counselling. For those who have ceased smoking, relapse prevention strategies. For patients unprepared or with no intention to change, counselling and intensive motivational interventions are recommended.
Alcohol	Combining therapeutic counselling by the family physician with multiple contacts, feedback, goal-setting, support at system level, particularly with regard to initial patient evaluation, and in some cases reminder or warning systems, provision of support materials. Motivational interviewing for alcohol dependents.
Multiple risk behaviours*	Evaluation of patient characteristics and needs and subsequent adaptation of intervention elements based on the evaluation, interactive education and skills promotion, self-monitoring, goal-setting, barrier identification and problem solving, use of multidisciplinary teams or nursing-based schemes and support systems in the centres such as reminder systems facilitating identification and multiple follow-up contacts.

* Based on the findings in secondary prevention studies, which may be generalised to the primary care context.

### Feasibility of integration of intervention strategies in PHC

The favourable opinion of the group with regard to integration of interventions targeting multiple risk behaviours was associated with concern about the actual possibility of integrating them into overburdened PHC centres. Experts considered that sustainable implementation of such interventions in the current PHC setting would require redesigning both centre organisation and intervention strategies. The need to overcome barriers related to (i) available resources, (ii) design of intervention programs and (iii) dissemination of these programs was raised (Table 3).

**Table 3:** Summary of proposals to enhance integration of healthy lifestyle promotion into PHC

1.- Increase availability of resources
• Increase the interaction time between patients and professionals in order to open their work agendas to health promotion:
- review care protocols for healthy people
- decrease checks for people with chronic diseases, promoting patient self-control and autonomy
- expressly prioritise health promotion activities and the reminding and recording of such activities
- effective administrative support to free practitioners from administrative and bureaucratic tasks
- communication, task redistribution, coordination and mutual support between physicians and nurses.
• Health policies defining the role of PHC in health promotion.
• Agreements between funding bodies and service providers specifically stating health promotion objectives, resources and indicators for evaluation.
• Participation of professionals in planning and quality evaluation of PHC services:
- promote communication within the health-care system
- establish common health promotion objectives for all professionals in the health-care organisation
- negotiate evaluation indicators shared by all groups.
• Actions at an inter-institutional level: town councils, schools, health-care centres, citizens' organisations, etc.
- designate a health promotion coordinator post at district or town level
- integrate initiatives and resources of the different sectors involved.

2.- Design of intervention programs
• Review their rationale based on scientific evidence of their effectiveness.
• Promote research into health promotion in PHC.
• Prioritise programs that are more flexible and adaptable to context.
• Participation of clinicians and researchers in the design and evaluation of new interventions.
• Use new support and reminder tools that do not interfere with the clinical practice of professionals.
• Take advantage of the new technologies for citizen information and education.

3.- Program dissemination
• Fight against resistance to change using outcome research.
• Set up a network of centres particularly interested in innovation for addressing multiple risk factors in PHC.
• Experience-based training and action-oriented skills.

i) Time is the scarcest and most needed of all **resources**. To gain time, changes in centre organisation and cooperation between professionals were proposed, including opening their work agendas to health promotion; reviewing care protocols for healthy people; decreasing the frequency of checks on people with chronic diseases, via self-monitoring and promotion of patient autonomy; expressly prioritising health promotion activities along with their reminding and recording systems; organising effective administrative support for PHC centres so as to decrease the time devoted by physicians to administrative tasks; and improving coordination, communication and mutual support between physicians and nurses, with the resultant redistribution of their tasks.

Other **financial and organisational resources **were considered to be required: establishment of health policies defining the role of PHC in health promotion at both individual and community level; the translation of such policies into program agreements between funding bodies and service providers that specify the health promotion objectives to be fulfilled, the resources needed to achieve them and the indicators for their evaluation; participation of professionals in the planning and evaluation of service quality; improved communication within the health system by establishing common health promotion objectives for all health-care organisation professionals, from the above-mentioned physicians and nurses to service management, including cooperation between health-care professionals and service users; and shared indicators for process and outcome evaluation to guide all actors in the same direction. In addition to these health-care resources, the group considered inter-institutional and inter-sector actions to be essential for linking health-care interventions to community resources and actions from town councils, schools, work centres, citizens' organisations and so on. Appointment of a coordinator for community-based activities and projects, who could be a town council social worker or educator, was considered to be necessary for this purpose.

With regard to ii), the **design of intervention programs**, the experts agreed that any efforts should be justified by scientific evidence that supports their effectiveness, as evaluated using appropriate instruments. The scarce scientific evidence available about the multiple risk factor approach makes new research on health promotion in PHC indispensable. However, while scientific evidence is necessary, it is not sufficient, as intervention programs must take into account the setting in which they are to be implemented and the unique characteristics of primary care. Intervention programs must therefore be flexible and adaptable. The importance of coordinated involvement of the various professionals working in PHC centres in the development and application of intervention strategies was emphasised, and constant reference was made to the supporting technological infrastructures. New, suitable information systems and recording and reminding tools that do not interfere with the work of professionals are required. Likewise, better use should also be made of both information-processing and dissemination resources. This includes using the waiting room to display information; using advertising techniques and local media; using information brochures and support materials; optimising current computing tools; and so on.

As regards iii), **program dissemination**, the introduction of new tasks into PHC appears to be rather more problematic due to a combination of the rigidity of the current health system, which does not allow for many changes, and the usual resistance to change of professionals, who are reluctant to modify their established routine. This particularly occurs when, as in this case, highly complex new interventions have to be incorporated. Outcome research was proposed as the method for promoting innovation in the area of health promotion, adoption of effective models or activities and exclusion of those that have not been shown to be effective. This requires the availability of a network of PHC centres with a special interest in integrating health promotion, where research programs for addressing multiple risk factors could be designed and implemented. At any rate, the need to redesign interventions and reorganise centres would not warrant a delay in implementation of strategies of proven effectiveness, although simultaneous research and action are required. Similarly, professionals should be trained to implement interventions whose effectiveness has been proven. This training should be aimed at achieving specific health promotion objectives and should be directed towards the teams of professionals who are supposed to implement interventions.

## Discussion

This research highlights the problems faced when attempting to integrate health promotion in PHC services. First, while useful theoretical models and relatively effective intervention strategies are available, they do not fit into the current PHC context. Second, the current organisation and resources in PHC centres, aimed almost exclusively at caring for disease, make implementation of such strategies difficult. As a result, effective strategies, such as counselling based on the 5-A's for smoking cessation and reducing alcohol intake, or physical activity prescription, are not routinely and widely implemented. Third, new interventions to effectively address multiple risk factors are required. Such interventions should integrate strategies such as the 5-A's with more intensive strategies, including motivational interviewing and a community-based approach, as well as resources within, and outside of, the health system. Fourth, sustainable and widespread implementation of strategies and programs for the prevention and promotion of healthy lifestyle behaviours in PHC has not been resolved and new ways to facilitate their integration should be investigated. In summary, the main reasons for the lack of sustainable integration of health promotion interventions in PHC include the selection of strategies that are not suited to the setting in which they are to be implemented, difficulties in changing clinical practice and service organisation, the lack of a socio-ecological approach that takes into consideration the multiple levels influencing healthy behaviour and inadequate use of methods for intervention design and evaluation [39,82-84].

In order to optimise the outcomes of health promotion interventions, their content and objectives should be adapted to the actual context where they are to be implemented [85-88]. To achieve this, the people who will be responsible for implementing interventions – the PHC professionals – should play a leading role in designing and evaluating innovative programs. These conclusions are consistent with recent initiatives in the area of translation of scientific evidence based on optimisation of clinical practice through research [85,86]. According to such models, research should be used to optimise clinical practice, instead of attempting to use the practice setting to show the relevance of prior studies. Thus, rather than researchers developing preventive interventions and then waiting for clinicians to incorporate them into their practice following a linear translation model, interventions should be designed in the context where they are to be implemented, with active involvement by the leading actors and for the purpose of adapting interventions to the requirements and characteristics of surgeries and centres [85-88]. Such optimisation processes should be multifaceted and should combine different strategies that have been shown to be effective in the field of innovation research, such as auditing and feedback, reminding systems or educational meetings of health-care professionals, and organisational interventions, such as review of the roles of professionals, creation of multidisciplinary teams, integrated care services, knowledge management, or quality management [89-92].

Sustainable implementation of effective interventions to address multiple risk behaviours will, however, require changes in PHC centres that allow for a flexible response to the new needs associated with the high prevalence of risk behaviours for chronic diseases and their prevention. This process will involve a mutual adaptation process between effective interventions and the operation of health-care centres. Models serving as a guide for health service change aimed at improving system quality are useful for such adaptation processes and are yielding promising results in addressing multiple risk behaviours in PHC [93-98]. These models identify the essential elements for providing high-quality health-care services, namely health-care delivery system redesign, based on a culture of quality improvement of the whole system, rather than merely focusing on the professionals working within the system; provision of support and resources for patient self-management; reorganisation of centres; the design of useful and efficient clinical information systems; support for evidence-based decision-making and enhancement of relationships with community resources [93,94].

Emphasising the influence of the community beyond the clinical setting is a critical issue for health promotion and disease prevention [95,97]. For health promotion strategies to be successful, they should be viewed from a socio-ecological perspective that jointly considers the multiple levels of influence on healthy behaviours – individual, family, community and society – as well as health-care and non-health-care resources [96]. Evaluation of the health status and characteristics of the population attending the centre is important in order to identify both the priority activities to be undertaken and the available resources in the community. Special attention should also be paid to simultaneous work on health promotion in a variety of settings, including schools, leisure and sports centres, health-care centres or town halls [99].

The greatest difficulty when implementing interventions aimed at the prevention and promotion of healthy lifestyles under current primary-care conditions may lie in the fact that these are complex interventions which comprise a great number of elements and target several interacting levels: the citizen as an individual, the health-care professional, working in a specific context of service provision to a community, within a particular health-care system [83,84]. There are multiple methodological challenges involved in the design, implementation and evaluation of such complex interventions, which makes a rigorous and systematic development framework to improve the effectiveness, impact and efficiency of such interventions, as well as their feasibility, indispensable. In this regard, the UK Medical Research Council's (MRC) taskforce on health-care services and public health defined a theoretical and methodological framework for the design and evaluation of complex interventions in clinical setting [83,84]. This framework, which uses qualitative and quantitative techniques simultaneously, comprises a number of phases, similar to the phases of clinical drug research, that may be implemented sequentially or iteratively: (a) a preclinical or theoretical phase for establishing the theoretical bases and identifying active components based on evidence; (b) a phase-I or modelling phase for component definition and identification of potential barriers to change and the mechanisms via which intervention should work; (c) a phase II or pilot trial to assess the feasibility and optimisation of the intervention and its evaluation; (d) a phase III or definitive randomised trial for controlled experimental evaluation of the intervention; and (e) a phase IV or long-term implementation under actual conditions. Several initiatives are currently successfully applying the MRC framework for the development and evaluation of complex interventions in PHC [100-102]. The study conclusions published to date agree on the value of the MRC framework as a tool for investigators to design, plan and assess innovative health-care and health promotion interventions in a clinical setting.

The results of our research correspond to the preclinical phase mentioned in the previous paragraph. It should be noted, however, that this study does not pretend to conduct an extensive review of Health Promotion or Health Education theories and models, but simply to facilitate discussion among the expert panel about the utility and feasibility of the main theoretical models for promoting healthy behaviour in primary care. We therefore restricted the review to the most commonly cited behaviour change theories in the primary care literature; other models aimed at planning health interventions, such as the PRECEDE-PROCEED model, or derived from the reviewed seminal models, were not used [38,39]. Our conclusions are limited by the composition of the expert panel, which excludes the viewpoints of health-care users, health-care managers and payers, as well as professionals from other non-health-care sectors. These results will have to be checked against the perceptions of these groups, although despite their limitations, they are still useful in terms of an initial approach to the problem and its potential solutions. Although the members of the expert group belong to the Basque Health Service, this service shares most characteristics with both the Spanish Health System and those of many other European countries. The study conclusions could therefore be generalized to the context of other similar PHC services across Europe. The conclusions of this research are also in agreement with those reported by other international groups and initiatives aimed at improving healthy lifestyle promotion in PHC [98,103-105].

## Conclusion

To address optimization of health promotion in PHC our team has designed an action research program, based on the present work, called "Prescribe Vida Saludable", which has the following strategic goal: to design, evaluate and transfer into routine PHC practice new feasible instruments, techniques and strategies that are effective for promoting physical activity, a healthy diet, smoking cessation and alcohol abuse avoidance.

## Competing interests

The authors declare that they have no competing interests.

## Authors' contributions

GG conceived the idea and is the study guarantor. He was primarily responsible for study design and planning, project coordination, supervision of the literature review, analysis and interpretation of results and manuscript preparation.

AS contributed to study design, was responsible for the literature review and collaborated in interpretation of results and manuscript preparation.

JMC collaborated in study design, obtained funding, coordinated the running of the discussion group sessions and participated in interpretation of results and manuscript preparation.

LB, CC, AA, IV and EM participated in analysis of results of the discussion groups and made a critical review of the manuscript.

All members of the "Prescribe Vida Saludable" group listed in the Acknowledgments section participated in the discussion group, collaborated in data collection and made a critical review of the manuscript.

All contributors approved this version submitted for publication to the ***BMC Health Services Research***.

## Pre-publication history

The pre-publication history for this paper can be accessed here:


